# Child and Adolescent Mental Health and Resilience-Focussed Interventions: A Conceptual Analysis to Inform Future Research

**DOI:** 10.3390/ijerph18147315

**Published:** 2021-07-08

**Authors:** Julia Dray

**Affiliations:** 1 School of Psychological Sciences, College of Engineering, Science and Environment, The University of Newcastle, Callaghan, NSW 2308, Australia; Julia.Dray@newcastle.edu.au; 2 Priority Research Centre for Health Behaviour, The University of Newcastle, Callaghan, NSW 2308, Australia; 3 Hunter New England Population Health, Hunter New England Local Health District, Wallsend, NSW 2287, Australia; 4 Hunter Medical Research Institute, New Lambton Heights, NSW 2305, Australia

**Keywords:** resilience, mental health problems, implementation, evaluation, children and adolescents

## Abstract

Internationally, the mental health of children and adolescents is undoubtedly an important construct of theoretical, clinical, and policy level concern. Worldwide, five mental disorders (depression, alcohol misuse, bipolar affective disorder, schizophrenia, and obsessive-compulsive disorder) represent half of the 10 leading causes of disability and premature death; with mental disorders accounting for 15–30% of disability adjusted life years in the first three decades of life. This provides a solid rational founded in implications for population health as to why reducing and preventing mental health problems in children and adolescents deserves attention. Past research has indicated interventions focussed on building resilience through strengthening protective factors may offer the potential to address mental health problems in children and adolescents, and in particular aid in reducing such problems during times of increased risk or adversity. With childhood and adolescence being critical periods of development, there is a need to reflect on the strengths and limitations of resilience-focussed interventions and anticipated future needs of the world’s youth. This conceptual analysis identifies a number of future research directions that may meaningfully add to the evidence base and improve implementation, evaluation, and impact of resilience-focussed interventions. These largely relate to refining the understanding of how resilience protective factors relate to mental health problems in children and adolescents. Important issues and potential opportunities to improve the related research field include improved reporting of intervention content; improved measurement of resilience protective factors in intervention trials; continued reporting and review of evidence of association between protective factors and mental health outcomes; and incorporation of mediation analysis within intervention trials. There is a need for further intervention studies in this space to be conducted as rigorous trials of resilience-focussed approaches based on such evidence of association, with clearly posited mechanisms of change, and inclusive of analysis of differential intervention effects. The suggested implications for research made in this conceptual analysis will aid in improving the quality of the evidence base relevant to the fostering of resilience and prevention of mental health problems in children and adolescents.

## 1. Introduction

Internationally, the mental health of children and adolescents is undoubtedly an important construct of theoretical, clinical, and policy level concern [1]. Based on data from 226 low-, middle-, and high-income countries, the WHO-GBD study in 2001 estimated mental disorders to account for 15–30% of DALYs in the first three decades of life [2]. Types of disability noted as associated with mental health problems in children and adolescents, and often persisting long-term, include: loss of productivity and contribution to the community; lower academic achievement; loss of well-being and quality of life; poor reproductive and sexual health; higher likelihood of engagement in health risk behaviours (including drug use and unsafe sex); and higher rates of mental stress, self-harm, and suicide [3,4,5,6,7,8]. This provides a solid rational founded in implications for population health as to why reducing and preventing mental health problems in children and adolescents deserves attention, including quality development of school-based interventions to improve mental health [6,7,9].

Worldwide, evidence varies in regards to whether prevalence of mental health problems in adolescents has increased in recent decades, and whether changes in prevalence are universal across all children and adolescents and mental health problems [10]. For example a UK review of adolescent mental health problems across a 25-year period found a general increase in prevalence of conduct and emotional problems in adolescents aged 15–16 years, across genders, all levels of social status and family type [11]. However a review of 19 studies across 12 countries found the results of studies to indicate a 30–50% increase in anxiety and depression only amongst adolescent girls, however, mixed findings for boys [12]. Currently emerging evidence from countries around the world suggests, since the advent of the COVID pandemic in 2019, children and adolescents are more likely to experience mental health problems, such as anxiety and depression (including during and after the pandemic) [13], with child and adolescent mental health problems identified as one of many priority areas for related action [14].

Many operationalisations of resilience exist [15,16]; however, it is often referred to as dynamic, and multifactorial, involving the ability to maintain or return to a positive state of mental health by employing multiple internal or external assets and resources (i.e., protective factors) [16,17,18,19,20,21,22,23,24,25,26]. A complex field, work in this space is diverse, with many prevention approaches adopted across relevant intervention studies internationally including: cognitive behavioural therapy; positive psychology; social and emotional learning; social skills; life skills; coping skills; interpersonal and self-management skills; psychological wellbeing therapy; the affective-behavioural-cognitive-dynamic (ABCD) model; mindfulness; and mental health promotion [27,28,29]. Whilst such approaches are based on many different intervention frameworks, they have been evaluated within school-based, intervention studies, internationally, and the commonality across these prevention interventions is that the strategies or intervention components all target the enhancement of protective factors central to the concept of resilience.

Past research has indicated interventions focussed on building resilience through strengthening protective factors central to the concept, may offer the potential for the prevention and reduction of mental health problems in children and adolescents [15,22,28,29,30,31], and in particular aid in reducing such problems during times of increased risk or adversity [25,29,32,33,34,35]. With childhood and adolescence being critical periods of development, there is a need to reflect on the strengths and limitations of resilience-focussed interventions. This conceptual analysis identifies a number of future research directions that may meaningfully add to the evidence base and support anticipated needs of children and adolescents, particularly in the current world context as the COVID-19 pandemic continues and following [13,25,36].

## 2. Refining the Understanding of How Resilience Protective Factors Relate to Mental Health Problems in Children and Adolescents

Many protective factors have been suggested to contribute to building resilience and/or preventing mental health problems among children and adolescents [17,19,22,31,37,38,39,40,41,42,43,44,45,46,47,48,49,50,51,52,53,54,55]. For example, Dray et al., 2017 [27], in undertaking a recent relevant systematic review of the related international evidence base, compiled a list of 31 internal and external protective factors from research published by eminent resilience and mental health researchers conducting eligible studies across 16 countries including: Australia, United States, Canada, Ireland, Germany, Italy, Netherlands, Scotland, Norway, China, Switzerland, England, Chile, India, Mauritius, and New Zealand [17,19,22,37,38,39,40,41,42,43,44,45,46,48,54,55] (Table 1). This list is not assumed to be complete or a ‘gold standard’, but does serve as a tool for classifying the multitude of factors targeted by resilience-focussed interventions. The review process highlighted the heterogeneity of factors targeted, with 26 protective factors (16 internal and 10 external) targeted across the 57 included trials—with up to 10 internal and 6 external factors targeted per included trial [27].

Other international experts in the field, such as Masten, et al., in the United States, have provided ongoing, and particularly recent, detailed explorations of advancements in the theory, methods, and knowledge of resilience-based research in children and adolescents (e.g., [23,25,45,50,56]). In one of their most recent papers, Masten and colleagues outline a shortlist of resilience factors reported across studies that span individual, family, school, community, and organizational domains [23] (Table 1). Similarly, in Australia a key agency for advancing mental health in Australia, Beyond Blue recently released a document to guide practitioners in building resilience in children [53]. A list of 24 factors considered to build resilience in children was developed from a non-systematic literature review of 13 key publications, and through consultation with practitioners, children, and parents [53]. The factors are described in domains referred to as ‘within the child’ (internal factors), ‘within the family’, ‘within the community and society’, and ‘within the family, community and society’ (external factors) (Table 1). In addition to the above mentioned list of factors, the recent Australian Beyond Blue publication provides a further listing of 31 factors, identified through a Delphi process with 25 experts, which are suggested to be those on which resilience programs should focus [53]. The 31 factors listed were those with at least 70% agreement between the 25 experts, 10 of which had 100% agreement (Table 1) [53]. However, the authors acknowledged a continuing inability to determine the relative merit or likely impact to be achieved through electing to focus on any one protective factor over another [53]. Taken together, the Dray et al. (2017), Masten (2021), and Beyond Blue (2018) publications are a sample of sources from recent years demonstrating the large variability in factors identified as relating to resilience across the related international evidence base.

To inform recommendations regarding which protective factors, from among such lengthy listings, should be the target of preventive interventions for mental health, there is also a need to understand the relative strength of their associations with mental health outcomes. While the WHO has suggested factors targeted in prevention programs should be evidence based [52], they provide little detail on the evidence base or process behind the selection of factors.

The following section examines a possible source of evidence around the strength of associations between protective factors and mental health outcomes; systematic reviews specifically examining associations between mental health problems and protective factors.

## 3. Evidence Contributed by Systematic Reviews Examining Associations between Mental Health Problems and Protective Factors

There has been an abundance of related systematic reviews published in recent decades (e.g., [23,57,58,59]); however, few have specifically attempted to consolidate evidence on associations between protective factors and mental health outcomes in children and adolescents [27,28,60,61,62,63]. A search for recent systematic reviews that had specifically attempted to consolidate evidence on associations between protective factors and mental health outcomes in children and adolescents, including reviews of descriptive data, was undertaken. Such a search could potentially assist with identifying key protective factors, alone or in combination. Three databases were searched, limited to the most recent decade (January 2011–present): PSYCInfo, Medline, and Embase. Combinations of keywords were used reflecting resilience (e.g., resilience, psychological, psychosocial factors), protective factors (e.g., protective, promotion, external, internal factors), mental health (e.g., mental health, mental disorders, mental illness, prevention, risk factors), and children and adolescents (e.g., adolescence, child). The search strategy was restricted to publication types of reviews, studies with humans, and published in English. This process resulted in the location of five relevant publications [27,28,60,61,62,63].

A summary of the mental health outcomes and factors examined in five such reviews, type of included studies, and the findings by method (i.e., meta-analysis or narrative synthesis of included study results) is shown in Table 2. Three reviews focussed on an outcome of depression, two of these examined evidence for a single protective factor (see Table 2). Stirling et al. (2015) [61] found no significant association between community connectedness and depressive symptoms [61]. Gariepy et al. (2016) found a significant association between low social support and depressive symptoms [62]. The third review, Cairns et al. (2014), indicated a sound or emerging evidence base for 18 protective factors, with significant but modest effect sizes indicated (see Table 2) [60]. Hence, across these reviews, for depression, evidence was provided for a number of factors aligning with those included in Table 1, including: positive and negative coping strategies [60]; relationships with positive peers [60]; self-disclosure to parents [60]; sport and physical activity [60]; and social support [62]. However, evidence was also provided for other factors outside of the framing of resilience presented in Table 1, such as: substance use, dieting, weight, sleep, dating during adolescence, and media use [60].

The fourth review, by Brumley and Jaffee, (2016), focussed on the outcome of externalising problems, used narrative synthesis, and indicated some evidence of association for 25 of 48 examined factors [63] (see Table 2). Evidence was indicated for a small number of factors that aligned with a resilience framework (according to the listings in Table 1) including: ability to refuse engaging in antisocial behaviour, family management and functioning, relationships with prosocial peers, and school attachment/connectedness [63]. However, evidence was also provided for other factors outside of this framing, such as: intelligence, sustained attention, verbal and visual memory, personality characteristics, house quality, and availability and exposure to illicit substances [63].

The fifth review, by Fritz et al. (2018), focused on psychopathology (depression, anxiety, internalising and externalizing, conduct disorders, PTS symptoms, emotional and behavioural disorders, and substance abuse), used narrative synthesis, and indicated some evidence for 13 of 25 individual-level factors, 6 of 12 family-level factors and 0 of 6 community-level factors [28] (see Table 2). Evidence was indicated for a number of factors that aligned with a resilience framework (according to Table 1) including: cognitive function, emotion regulation, social interaction/attachment, personality/self-concept, family support, parental support, social support [28].

A sixth review by Dray et al., 2017 [27], did not specifically focus on whether the targeted protective factors were measured or changed in response to the intervention, however these data were extracted [27]. Of 57 trials, 37 (65%) included a measure of at least one protective factor (see Table 3); and of these 37 trials, 21 (57%) reported change in at least one factor. Eighteen of 37 trials (49%) had a positive impact on at least one mental health outcome, with 14 (78%) of these also showing a positive effect on at least one protective factor [27]. However, protective factors targeted by the intervention did not always align with those measured in the trial and, overall, trials only measured a portion of all targeted protective factors (see Supplementary Table S1). The overview outlined in Table 3 may, at least indirectly, suggest a higher likelihood of positive intervention effect on mental health outcomes when a positive change in protective factors is also achieved.

Further, in the Dray et al. review, of the 37 included trials that measured protective factors, 2 trials [64,65] included a mediation analysis (Table 4). In intervention research, mediation analysis can enable the investigation of whether factors considered causally linked to an outcome, and therefore targeted in an intervention, do mediate the intervention effect on the dependent outcome as hypothesised [66]. As such, mediation analysis allows investigation of ‘how’ interventions work [66]. The positive results of the mediation analysis in one of the two trials [64] reinforces the potential for change in protective factors to mediate change in mental health outcomes. Finally, evident when comparing Table 1 and Table 2, whilst all sources of evidence reviewed can be grouped within a mental health context, terminology used to identify seemingly similar factors varies (e.g., family cohesiveness vs. family cohesion, delinquent peer affiliations vs. relationships with prosocial peers, social connectedness vs. social support), as does domains used to organise factors (e.g., internal, external, individual, family, peer, school, neighbourhood, social). Such differences in terminology introduce a need when comparing sources of evidence to apply a level of subjectivity in order to compare potentially ‘like’ factors, and introduces an added layer of difficulty in drawing conclusions.

In summary, the brief review of various sources of evidence undertaken here highlights an extensive list of factors. From systematic reviews, evidence of association was identified between mental health outcomes and some factors that aligned with the lengthy listings of resilience protective factors outlined in Table 1, as well as some outside of lists developed from a resilience protective factor framework. Reviews that include quantitative synthesis for a range of factors and grade level of evidence appear of particular value for identifying factors with the strongest evidence-base to target in preventive interventions. Additionally, this preliminary investigation highlighted the need to, and value in, measuring all targeted protective factors in future research. While such comprehensive measurement may need to be weighed against burden on participants, if feasible, it could potentially progress knowledge of the mechanism of intervention effects considerably (e.g., through mediation analyses). Further application of mediation analysis in future trials, inclusive of mediation analyses for a larger range of protective factors and mental health problem outcomes, is likely to provide a stronger sense of whether protective factor change drives intervention effects on mental health outcomes, and be helpful in identifying critical elements for inclusion in resilience-focussed programs. Greater consistency in terminology for seemingly similar concepts, whilst difficult, could make research comparison and synthesis easier.

## 4. Discussion

This conceptual analysis explored key concepts and issues relating to resilience focussed interventions for child and adolescent mental health problems, some elements of each concept, and the connections between them. It is intended to extend current knowledge relating to resilience-focussed interventions for child and adolescent problems, by presenting a novel argument, interpretation, and critique of the existing research evidence, and suggestions on future directions required to refine such evidence. International sources of evidence were examined in this conceptual analysis, including a sample of recent peer review publications demonstrating the large variability in factors identified as relating to resilience and systematic reviews that had specifically attempted to consolidate evidence on associations between protective factors and mental health outcomes in children and adolescents in the past 10 years.

This conceptual analysis highlights that there is a need to conduct quality intervention trials to optimally inform the field. Evidence suggests that across child and adolescent trials collectively, universal programs that target protective factors can have a positive impact on mental health outcomes [27]. It seems likely, and perhaps not inappropriate, that there will continue to be growth in the number of trials being undertaken using this approach. The context within which future trials will be undertaken is one where education frameworks and mental health policies in Australia [67,68] and internationally [69,70,71] already recommend universal preventive programs to target a range of factors to promote mental health for children and adolescents within the school setting. Such recommendations are being actioned and there is a range of programs to promote mental health currently in place in schools [72,73,74,75], with some explicitly incorporating building resilience [68,70,76,77,78,79].

Given the likely expansion of research in this area and the ongoing implementation of programs in school settings to improve mental health, there is an imperative to ensure future trials or program evaluations are of sound quality in order to optimally inform the field. A number of considerations and recommendations pertaining to the conduct and methodological rigour of future trials, evident from this conceptual analysis, are outlined below.

### 4.1. Provide Clarity and Rationale for Conceptual Underpinning and Intervention Content

It is apparent that there is no single, common understanding of what constitutes a ‘resilience intervention’ per se. Few of the trials explicitly state that the intervention is resilience-focussed, and the factors targeted in those trials that identify resilience as their frame are not readily distinguishable from those that do not [27]. The body of literature reporting on trials that have targeted multiple protective factors, in an array of combinations, is large. A recent attempt within the Australian context to synthesise the ‘evidence’ from literature and expert opinion in order to provide guidance for ‘practice’, concludes that there is no (or little basis) on which to recommend one particular factor, or combinations of factors, as necessarily more important to focus on compared to others [53]. The above brief attempt to identify such evidence within previous international systematic reviews and trials found somewhat stronger evidence for a small range of factors compared to others, however ultimately noted limitations of the current evidence-base, still largely allowing no concrete recommendations regarding what protective factors may enable greatest impact on particular child and adolescent mental health problems. In general, many trials did not provide a clear rationale for the chosen conceptual underpinning, nor a rationale or evidence-base for the protective factors chosen for targeting within the intervention. The clarity and quality of literature in the field would be improved if authors specified the protective factors being targeted in the intervention and the rationale and evidence-base for doing so, including specification as to whether change in protective factors is the proposed mechanism for change in mental health.

In addition, when designing future interventions, it is important to consider who will be the ‘driver’ of intervention implementation. Strategies are emerging for supporting schools to lead and sustain implementation [80], and education and funding bodies are increasingly interested in applicability of programs to ‘real world’ contexts [81]. It has been suggested that pragmatic approaches may offer advantages including the potential to better tailor interventions to local needs [82], and allow flexibility in implementation to enable integration of intervention strategies and research evaluation with normal school practices and support sustainability [83]. However, such approaches may also entail challenges, such as difficulty in sufficiently defining interventions so that there is a clear understanding of the expected intervention content and intensity of delivery [82,83]. There have been relatively few trials that have utilised a pragmatic approach, and further research is required to allow a considered assessment of their relative advantages and disadvantages [81].

### 4.2. Ensure Sound Resilience Protective Factor Measurement

There is value in ensuring that measures of resilience protective factors are included at baseline and all subsequent follow-ups if possible, and that the protective factors measured align with those targeted in the intervention. Further, it is important to consider whether the protective factor measure has reasonable psychometric qualities. In a 2011 systematic review, Windle et al. examined the psychometric properties of 15 resilience measurement scales. Six of fifteen scales were developed for use with children and adolescents, and received low ratings for psychometric quality [84]. Only five of fifteen measures (including four of the six developed for children and adolescents) examined protective factors related to resilience across multiple levels (e.g., personal/individual, family and community) [84]. Data regarding sensitivity to change were available for only one of fifteen measures (zero of six child and adolescent measures) [84]. The development of tools to more accurately and reliably measure resilience protective factors—and to measure change in such factors within intervention trials—would be beneficial [19]. Similarly, in a 2020 psychometric meta-analysis, Renbarger et al., examined the cross-cultural utility of the Child and Youth resilience Measure (CYRM) [85]. Whilst the tool has been used globally, the review found that few studies reported reliability or validity of the measure [85]. There remains a need to establish and utilise robust resilience protective factors in resilience-focussed intervention studies.

### 4.3. Examine Differential Intervention Effect for Subgroups

Additionally, as noted, there are many differences in, and levels of experience of, challenges and adverse outcomes linked to mental health problems in children and adolescents, including: loss of productivity and contribution to the community; lower academic achievement; loss of well-being and quality of life; poor reproductive and sexual health; higher likelihood of engagement in health risk behaviours; and higher rates of self-harm and suicide [3,4,5]. Similarly, there are many differences in the prevalence of specific mental health problems in children and adolescents such as by sociodemographic characteristics (for example: age and gender [86]). Conducting moderator analysis allows investigation of who the intervention may provide greatest preventive benefit to, as well as whether the intervention may have unforeseen adverse effects for some subgroups of the target population [87]. In future intervention studies, it may be helpful to investigate intervention effects (on both mental health outcomes and protective factors) for subgroups within the target population. This may have particular benefit for those identified in previous research as experiencing inequity in the prevalence of key outcomes of interest.

Some reviews (e.g., [27]) have endeavoured to complete subgroup analysis by sociodemographic characteristics however attempts to do so have been hampered by lack of investigation of differential intervention effects by factors such as age and gender in relation to both resilience protective factor and mental health problem outcomes in relevant past individual studies. For example, of the 57 trials included in the Dray et al., review [27], three reported findings of a moderator analysis [88,89,90], investigating differences in intervention effect on mental health outcomes by baseline level of protective factors. Two trials included the outcome of depressive symptoms [88,90], and the third trial included both anxiety and depressive symptom outcomes [89]. Across these trials: students with poorer family functioning/relationships (external factors) at baseline showed greater reductions in anxiety and depressive symptoms at follow-up [89]; students with lower self-efficacy (internal factor) at baseline showed greater reductions in depressive symptoms at follow-up [90]; and in the third trial, no significant results were found for the factors of self-efficacy, optimism, and coping (internal factors) in relation to depressive symptoms [88]. Such results attest to the potential for differential intervention effect and demonstrate potential for increased richness of data when subgroup analyses are incorporated. Conducting moderator analysis in future trials will require their incorporation in a priori power calculations. While low frequency counts for some groups at individual trial level may potentially restrict completion of moderator analysis or render them as exploratory, where possible reporting of such subgroup data offers potential for trials to contribute to meta-analysis of intervention effect by subgroup in systematic reviews. Should more evidence of this nature become available, an important expansion of the current conceptual analysis will be to conduct a comprehensive examination of this, provide related synthesis, analysis and interpretation of such findings, and subsequent helpful recommendations for the field.

In summary, there are a number of considerations and recommendations pertaining to the conduct and methodological rigour of future trials. Whilst many intervention trials have been (and likely will continue to be) undertaken in this area, it is important that they are undertaken utilising rigorous methodology if knowledge in the field is to take substantive steps forward. The particular issues and recommendations discussed above—including clarity around underpinning conceptual issues, sound measurement of resilience protective factor outcomes, and incorporation of sub-group analyses where possible—represent some important issues to consider and potential opportunities for improvement.

### 4.4. Limitations of the Current Conceptual Analysis

This conceptual analysis should be considered in light of some limitations including: provision of a select, but quality and reputable, sample of sources listing factors identified as relating to resilience, and the inclusion of a systematic search strategy as one potential source of evidence, however, not conducted as a full systematic review and only focussing on the most recent decade of systematic reviews examining associations between mental health problems and protective factors. Whilst outside the scope and purpose of this conceptual analysis, both sources of evidence could be taken and strengthened into full systematic or rapid reviews, to aid in progressing the opportunities for improvement to the field identified here.

## 5. Conclusions

Overall, application of the concept of resilience within the school setting in recent decades, and the expansion of the related research field, suggests likely continued growth in the development and implementation of universal, school-based, resilience-focussed interventions, with the possibility of larger scale rollouts. Given this, it is important that researchers work towards being able to accurately measure mental health in children and adolescents, to aid continued population level monitoring of mental health, and robust measurement in research trials. To better inform intervention content, it is important that researchers work towards improving the understanding of the relationship between protective factors and child and adolescent mental health outcomes, through: improved measurement of protective factors in intervention trials; continued reporting and review of evidence of association between protective factors and mental health outcomes; and incorporation of mediation analysis within intervention trials. There is a need for any further intervention studies in this space to be conducted as rigorous trials of resilience-focussed approaches based on such evidence of association, with clearly posited mechanisms of change, and inclusive of longer-term follow-ups and analysis of differential intervention effects. Considering further development and evaluation of pragmatic interventions may be worthwhile to further understand the impact of this approach. The suggested implications for research made in this conceptual analysis will aid in improving the quality of the evidence base relevant to the fostering of resilience and prevention of mental health problems in children and adolescents.

## Figures and Tables

**Table 1:** Sample of sources demonstrating the large variability in factors identified as relating to resilience.

Factors for Resilience Utilised in Dray et al., 2017, Review of International Peer Review Publications [27]	Factors Identified by Masten et al., 2021, as a Short List of Many Reported in Resilience Studies [23]	Factors that Build Resilience Identified by Beyond Blue, Literature and Consultation Based, 2018 [53]	Factors Identified by Beyond Blue for Focus in Resilience Interventions Developed from Expert Consensus, Using a Delphi Process [53]
Internal protective factors Cognitive competence Cooperation and communication Coping Emotional regulation Empathy Empowerment Goals and aspirations Moral competence Problem solving/decision making Spirituality Self-control Self-efficacy Self-esteem Self-regulation Self-awareness Social and emotional competence Social and emotional skills External protective factors Community adult high expectations Community caring relationships Community meaningful participation Community support Home adult high expectations Home caring relationships Home meaningful participation Home support Peer caring relationships Pro-social peers School adult high expectations School caring relationships School meaningful participation School support	Factors noted as reported in resilience studies across individual, family, school, community, and organisational levels: Sensitive caregiving, close relationships, social support Sense of belonging, cohesion Self-regulation, family management, group, or organization leadership Agency, beliefs in system efficacy, active coping Problem-solving and planning Hope, optimism, confidence in a better future Mastery motivation, motivation to adapt Purpose and a sense of meaning Positive views of self, family, or group Positive habits, routines, rituals, traditions, celebrations	Factors within the child Genetic and biological factors Positive self-esteem Positive self-talk/self-compassion Autonomy/independence Ability to identify/articulate emotions Self-regulation Confidence/self-competence Social skills and empathy Optimism/positive attitude Sense of responsibility/connection to family/community Healthy thinking habits Factors within the family Positive family relationships Family identity/connectedness Effective parenting Factors within the community and society Peer connections/interactions Education settings providing positive encouragement/positive relationships Socially inclusive, and family/child friendly community values/beliefs Socially inclusive, and family/child friendly spaces Socially inclusive, and family/child friendly public policies Factors within family, community, and society Opportunities for healthy risk taking Opportunities for positive experiences Ongoing, meaningful relationships with ≥1 significant supportive adult who the child can talk to (e.g., family member, teacher, community mentor)	Factors with 100% consensus Family cohesion Family environment Peer relationships Pro-social skills and empathy Positive coping skills Self-regulation Sense of agency Self-reflection Problem-solving Self-efficacy 91–99% consensus Social connectedness Extra familial support (sports/community groups) Planning Self-compassion Sense of meaning and purpose Optimism Hope Positive emotional experiences Environmental resources 81–90% consensus Sibling relationships Cultural connectedness Locus of control Sense of coherence Mindfulness Perseverance Mastery experiences Cultural identity 71–80% consensus Mentors Humour Physical activity Talents and interests

**Table 2:** Systematic reviews examining associations between mental health problems and protective factors.

Review/Mental Health Outcome	Type and Age Range of Included Studies/Analysis	Individual Level Factors	Family Level Factors	School or Peer Level Factors	Community or Neighbourhood Level Factors
Stirling et al., 2015 [61] Depression	18 cross-sectional and 3 cohort studies, children and adolescents (4 to 18 years); meta-analysis, protective factor	Community connectedness ^N^			
Gariepy et al., 2016 [62] Depression	18 cross-sectional and 13 cohort studies, children and adolescents (8 to 20 years); meta-analysis, protective factors				Low social support **
Cairns et al., 2014 [60] Depression	69 prospective cohort studies, adolescents (12 to 18 years); meta-analysis, evidence for risk and protective factors summarised into sound, emerging, minimal or insufficient (indicated by subscript of ^1, 2^ or ^3^ respectively)	Positive coping strategies **^,1^ Negative coping strategies **^, 1^ Alcohol use (frequency) **^, 1^ Alcohol use (quantity) **^, 1^ Cannabis use **^, 1^ Dieting **^, 1^ Healthy dieting ** Other illicit drug use **^, 1^ Polydrug use **^, 1^ Sleep **^, 1^ Tobacco Use **^, 1^ Weight **^, 1^ Physical activity **^, 2^ Media use **^, 2^ Private religious observance ^3^ Positive emotion regulation strategies ^3^ Negative emotion regulation strategies ^3^ Early sex ^3^	Pelf-disclosure to parents **^, 2^ Early moving out ^3^	Relationships with positive peers **^, 2^ Extra-curricular activities^N,3^ Sport **^, 2^ Dating during adolescence **^,2^	Public religious observance ^3^ Part-time employment ^3^
Brumley and Jaffee, 2016 [63] Externalising problems	60 longitudinal studies, children and adolescents (year range not provided). Narratively summarised quantitative association analysis results of included studies, protective factors	Self-esteem/self-confidence Self-regulation Interpersonal callousness * Ability to refuse engaging in antisocial behaviour * Involvement in prosocial activities Attitudes toward delinquency * Intelligence * Academic achievement Academic aspirations for higher education * Sustained attention * Delayed verbal memory * Delayed visual memory * Attention problems * Difficult temperament/surgency * Easy temperament/effortful control * Shyness * Depression *	Family management * Family functioning * Family cohesiveness Parental stress * Parent-child relationship quality Attachment/closeness to parent Perceived acceptance by parent Positive parenting (global measure) Parental warmth Parental sensitivity Parental empathy Parental monitoring Parental responsiveness Parental involvement Parental supportiveness Parental overprotection * Parent disapproval of antisocial behaviour Maternal socialisation of coping Maternal self-esteem Grandmother involvement	Delinquent peer affiliations * Relationships with prosocial peers * Ability to get along with peers * Well-liked by peers School protective factors School attachment/connectedness * Attitudes towards school * School commitment	Social cohesion Collective efficacy Housing quality * Community crime Perceived availability and exposure to marijuana *
Fritz et al., 2018 [28] Psychopathology	22 cohort studies, adolescents and young adults (13–24 years).Narrative described moderating or mediating resilience factors of included studies	Cognitive function (high: cognitive reappraisal, mental flexibility; low: rumination) Emotion regulation (high: distress tolerance; low: alcohol coping expectancy, aggression, expressive suppression) Social interaction/attachment (low: insecure attachment, disconnection/rejection, other-directedness) Personality/self-concept (high: self-esteem high self-efficacy; low: ego over-control, ego under-control)	Family support (high: family cohesion, positive family climate, immediate family support, extended family support) Parental support (high: positive parenting, parental involvement)		Social support

^N^ Factors for which meta-analysis indicated no significant association with mental health outcome; ** factors identified as related to mental health outcomes using meta-analysis; * factors for which some evidence of association with mental health outcomes was noted using narrative summary of included study results. ^1,2,3^ For the meta-analysis performed by Cairns et al., 2014 [60], evidence for risk and protective factors was summarised into sound, emerging, minimal or insufficient and is indicated by subscript of ^1, 2^ or ^3^ respectively.

**Table 3:** Summary of results of included trials from the Dray et al. systematic review that included a measure of protective factors (PFs) [27].

	Positive MH ^a^ n	Null MH ^b^ n	Total n (%)
Positive PF ^c^	14	7	21 (57)
Null PF ^d^	4	12	16 (43)
Total n	18	19	37 (100)

^a,b^ Positive or null intervention effect relative to a control or alternate intervention deemed ‘equivalent’ to a control, for measured mental health outcome(s), respectively. ^c,d^ Positive or null intervention effect relative to a control or alternate intervention deemed ‘equivalent’ to a control, for at least one measured protective factor, respectively. MH: Mental Health Outcome

**Table 4:** Summary of the Dray et al. systematic review [27] included trials that incorporated mediation analysis.

Study	Mental Health (MH) Outcome	Summary of Intervention Effects	Protective Factors (PFs) Targeted	PFs Included in Mediation Analysis	Mediation Analysis Results
Essau 2012 [64]: Sample size: n = 638 Mean age: 10.9 years Intervention length: 26 weeks	Anxiety symptoms	MH Significant intervention effects for anxiety symptoms at post-intervention, 6- and 12-month follow-up. PFs Significant intervention effects for perfectionism, coping, and social and adaptive functioning; however, not for social skills.	Empathy, cognitive competence, coping, problem solving/decision making, goals and aspirations.	Perfectionism * Coping Social skills * Social and adaptive functioning *	Perfectionism and coping acted as mediators of change in pre- to post-test anxiety symptom scores. Non-significant for social skills and social and adaptive functioning.
Horowitz 2007 [65]: Sample size: n = 380 Mean age: 14.43 years Intervention length: 8 weeks	Depressive symptoms	MH Positive intervention effect for depressive symptoms at post-intervention, not sustained at 6-month follow-up. PFs Significant intervention effect for cognitive competence. No significant effects for coping and quality of parent-child relationships.	Goals and aspirations, cognitive competence, problem solving/decision making, coping.	Attributional style (cognitive competence) Coping Quality of parent-child relationships *	Non-significant results for mediation analysis.

* Measured and included in mediation analysis; however, not targeted in intervention.

## Data Availability

Data sharing not applicable.

## Supplementary Materials

The following are available online at https://www.mdpi.com/article/10.3390/ijerph18147315/s1, Table S1: Mapping of targeted protective factors against measured protective factors, for 37 trials included in the Dray et al., review incorporating a measure of protective factors [27].
