# Supplementary material for: Child and Adolescent Mental Health and Resilience-Focussed Interventions: A Conceptual Analysis to Inform Future Research
Source: Int J Environ Res Public Health. 2021 Jul 8;18(14):7315. doi: 10.3390/ijerph18147315 (PMC8303353; doi:10.3390/ijerph18147315)
Supplement: Supplementary file 1 [file ijerph-18-07315-s001.zip › ijerph-1249011-supplementary.pdf]

Supplementary Table S1: Mapping of targeted protective factors against measured protective factors, for 37 trials included in the Dray et al., review incorporating a measure of protective factors[36]

**Resilience Protective Factors (PF)**	**n trials that targeted PF**	**n trials that measured PF**
Internal resilience protective factors		
Cognitive competence	23	19
Cooperation and communication	23	1
Coping	18	12
Emotional regulation	8	3
Empathy	16	3
Goals and aspirations	8	0
Problem solving/decision making	28	2
Self-control	3	0
Self-efficacy	6	5
Self-esteem	9	2
Self-regulation	19	2
Self-awareness	6	1
Social and emotional competence	22	6
Social and emotional skills	13	7
Spirituality	1	0
External resilience protective factors		
Community caring relationships	0	1
Community meaningful participation	2	1
Community support	5	2
Home caring relationships	4	4
Home support	3	0
Peer caring relationships	9	2
Pro-social peers	3	2
School caring relationships	3	3
School meaningful participation	5	5
School support	3	2

NB: No included trials targeted or measured 6 of thirty-one factors utilised in the review. These were 2 internal factors: empowerment and moral competence; and four external factors: community adult high expectations, home adult high expectations, home meaningful participation and, school adult high expectations.
